# 

**Published:** 1799-10

**Authors:** 


### NEW MEDICAL PUBLICATIONS IN SEPTEMBER.

*The first volume of the Medical and Physical Journal, containing the earleist information on subjects of Medicine, Surgery, Pharmacy, Chemistry, and Natural Philosophy; together with a Critical Retrospect of all new books in these departments of literature.* Conducted by T. Bradley, M. D. and A. F. M. Willich, M. D. 8vo. 10s. boards. *Phillips.*

*A Treatise on Febrile Diseases, including intermitting and continued fevers, eruptive fevers, inflammations, hæmorrhages, and the profluvia.* By A. P. Wilson, M. D. 8vo. 9s. boards. *Cadell* and *Davies.*

*A Synopsis of the Chemical Characters. adapted to the new Nomenclature proposed by Messrs. de Morveau, Lavoisier, Bertholet, and De Fourcroy,* &c. &c. By W. Jackson, *Practical Chemist.* On a whole sheet copper-plate. *Price* 2s. plain, and 2s. 6d. coloured. *Symonds.*

### NEW MEDICAL PUBLICATIONS IN GERMANY.

*Anatomische Tafeln:*—Anatomical Tables, Part VI. Numb. I. Containing Angiology; with a Latin and German Text, (4 rix-doll. Sax. Curr. or about 15s. British) *Weimar.* Board of Industry.

*Disquisitio botanico-mediea Tremellæ Nostoch: cui accedit Tremellæ palmatæ descriptio,* c. tab. aen. 4to. *Lipsiæ.* Barth.

*Einschraenkuengen, &c.* Strictures on the latest Essays relative to Brown's Theory of Excitement: By F. W. C. Hunnius, M. D. 8vo. (16 grosch. or about 2s. 1d.) *Weimar* Gœdicke.

---

### TO CORRESPONDENTS.

*We have received* two *communications relative to the diſcovery of muſk and ſalt of hartſhorn, in gangrene and ſphacelus. As, however, this ſubject appears to us* nearly *exhauſted, we are under the neceſſity of deferring them to a future Number, while we gratefully acknowledge the favours of our Correſpondents.*

*The following papers have alſo been received, and ſhall, if room permit, be inſerted in the next Number: "A favourable termination of an adheſion of the placenta after delivery."—"Obſervations on one of the means by which the eye has been ſuppoſed to accommodate itſelf to the different diſtances of objects."—"An Analyſis of the Inſtitutions of Practical Medicine, delivered in Lectures: By* J. B. Burserius de Kanifeld, *&c.*"

*The "Queſtions" addreſſed to us by our obliging Correſpondent from Kidderminſter, we muſt decline to anſwer; as they do not appear to involve a propoſition connected either with medicine, or its "uſeful" auxiliary branches.*

*The anonymous letter from Stourbridge, addreſſed to Dr. W. was ſo complete a ſpecimen of infamy, that it has been returned to the General Poſt Office, for the recovery of the poſtage; eſpecially as the writer had the additional indiſcretion to encloſe it in a* double *cover, and to direct it to the care of a reſpectable bookſeller in town.*

